# Trace element accumulation in *Salvinia natans* from areas of various land use types

**DOI:** 10.1007/s11356-019-06189-5

**Published:** 2019-08-18

**Authors:** Ludmiła Polechońska, Agnieszka Klink, Małgorzata Dambiec

**Affiliations:** Department of Ecology, Biogeochemistry and Environmental Protection, University of Wrocław, ul. Kanonia 6/8, 50-328 Wrocław, Poland

**Keywords:** Floating fern, Water pollution, Phytoremediation, Bioindicator, Self-organising feature map (SOFM), Neural network

## Abstract

*Salvinia natans* meets many criteria for accumulative bioindicators and phytoremediation agents. However, the majority of studies on its bioaccumulation capacity were performed under controlled culture conditions. In the present study, *Salvinia natans* was investigated in a field study. Plant and water samples were collected from aquatic reservoirs located in areas with various dominant land uses (forested, agricultural, residential and industrial). Contents of 10 trace elements (As, Cd, Co, Cr, Cu, Fe, Mn, Ni, Pb, Zn) and phytomass were measured to estimate the bioindication and phytoremediation potential of the species. Results showed that contents of trace elements in *S. natans* were high compared with other aquatic ferns (*Azolla japonica*, *A. pinata*) as well as free-floating vascular plants (e.g. *Pistia stratiotes*, *Hydrocharis morsus-ranae*, *Lemna* sp., *Eichhornia crassipes*)*.* High bioaccumulation factors for Cu, Fe, Mn, Ni, Pb and Zn confirm accumulative abilities of the plant. Application of neural networks (SOFMs) confirmed that the species may be used in bioindication: the land use type determined the composition of substances carried into water reservoirs with runoff and trace elements accumulated in *Salvinia* tissues. Ferns in industrial areas had the highest content of Cd, Cu and Zn, while in residential areas plants showed the highest content of As, Co, Fe, Mn, Ni and Pb. Element contents in *S. natans* in forested areas were the lowest. High standing stocks of Cd, Mn and Ni indicated an important role of *S. natans* in the cycling of elements and potential use in their removal from aquatic ecosystems.

## Introduction

Due to the growing deficiency and contamination of waters, increasingly more attention is paid to pollution monitoring techniques and alternative methods of decontamination. Bioindication is a promising method which provides information on the degree of pollution or degradation of ecosystems by observation of the representative organisms that contain information on the quality of the surrounding environment (Markert et al. 2003). Phytoremediation has been recommended as a cheaper and effective alternative to conventional (physical and chemical) methods for the removal of toxic elements (Dhir and Srivastava 2011). Both these techniques are based on the natural ability of organisms to accumulate, store and biodegrade contaminants within their tissues. Many aquatic macrophytes have shown a high capability to accumulate trace elements from water or bottom sediments (Dhir et al. 2009), but they differ in both the intensity of accumulation in their tissues and in biomass (Rezania et al. 2016). Therefore, the suitability for bioindication and effectiveness of phytoremediation are strongly correlated with the selection of an appropriate plant species (Hołtra and Zamorska-Wojdyła 2014). An accumulative bioindicator should be easy to identify in the field, easy to collect and handle and abundant and should show correlation between the environmental concentration of the pollutant to be monitored and the concentration in the organism (Brooks and Robinson 1998; Markert et al. 2003). An ideal phytoremediation agent should have ability to tolerate and accumulate large amounts of potentially toxic trace elements in its easily harvestable parts as well as have high biomass production and a fast growth rate (Goswami and Das 2015).

The species under study, *Salvinia natans* (L.) All., meets most of these criteria: it is easy to identify, grows rapidly, easily spreads and is easy to harvest (Dhir et al. 2011; Szmeja and Gałka 2013). It shows wide geographical distribution within temperate, tropical and sub-tropical regions (Dhir et al. 2009; Szmeja and Gałka 2013), which is important because local plant species are better in terms of survival, growth and reproduction under some environmental stresses and using them for phytoremediation is more effective (Kamran et al. 2014). Moreover, several *Salvinia* species were shown to accumulate large quantities of trace elements in their tissues, including As, Pb (Hoffmann et al. 2004), Cd, Cu (Buta et al. 2014) and Cr (Dhir et al. 2009). Contaminant uptake in *Salvinia* occurs through a biological or physical mode. Metal (e.g. Cr, Pb) uptake by physical processes is fast and involves adsorption, ionic exchange and chelation, while biological processes such as intracellular uptake (transport through plasmalemma into cells) are comparatively slow but aid in subsequent translocation of metals (e.g. Cd) from submerged leaves to floating ones (Suňe et al. 2007).

Although extensive literature highlights the ability of several *Salvinia* species for trace element accumulation from multi-metal solutions or wastewater (Buta et al. 2014; Das and Mazumdar 2016; Dhir and Srivastava 2011), the majority of studies were performed under controlled culture conditions. The metal accumulation efficiency of the species in a field experiment in aquatic ecosystems has not been addressed till date. Therefore, the aim of the present study was to evaluate the content of trace elements in *S. natans* growing in natural populations in areas of various land use types (forested, agricultural, residential and industrial) and to evaluate its bioaccumulation efficiency, the features important in assessing its suitability for bioindication and phytoremediation.

## Materials and methods

### Species description

*Salvinia natans* is a floating aquatic fern from the *Salviniaceae* family. The species is widely distributed: it is native to central and southeastern Europe and the major part of Asia. It is also found in few localities in North Africa (i.e. in Algeria) and North America (Szmeja and Gałka 2013). *S. natans* inhabits shallow reservoirs with eutrophic water and fertile bottom sediments. It has slender branching stems and three leaves in the whorl. Two leaves are floating, oval, about 10–14 mm long and 6–9 mm wide, and covered with papillae. The third, submerged leaf is ultimately divided and root-like (Piękoś-Mirkowa and Mirek, 2003; Tutin et al. 1980). The species reproduces by sexual and vegetative means. Sporophytes have a clonal structure built by adding new modules that grow like lateral branches in vascular plants. Fast production of new modules as well as an increase in the number and size of ramets in the modules contributes to the fast clonal expansion of *S. natans.* Sporocarps, formed from August to the beginning of November on submerged leaves, overwinter at the bottom of water reservoirs, and in spring (usually March to May), they release spores that develop into new plants. Sporocarp emergence and production are regulated by population density, temperature and evapotranspiration (Coelho et al. 2005; Szmeja and Gałka 2013; Zutshi and Vass 1971). The species can double its biomass in a time span of 2 days and form dense populations (up to 320 specimens per m^2^) that sometimes cover the entire water surface (Dhir and Srivastava 2011; Piękoś-Mirkowa and Mirek, 2003). Although the species is abundant and widespread, it is protected in Poland and was classified as least concern (LC) in the IUCN Red List of Threatened Species due to naturally occurring massive population fluctuations and local destruction of habitats (Allen 2011).

### Study area

**Fig. 1:**
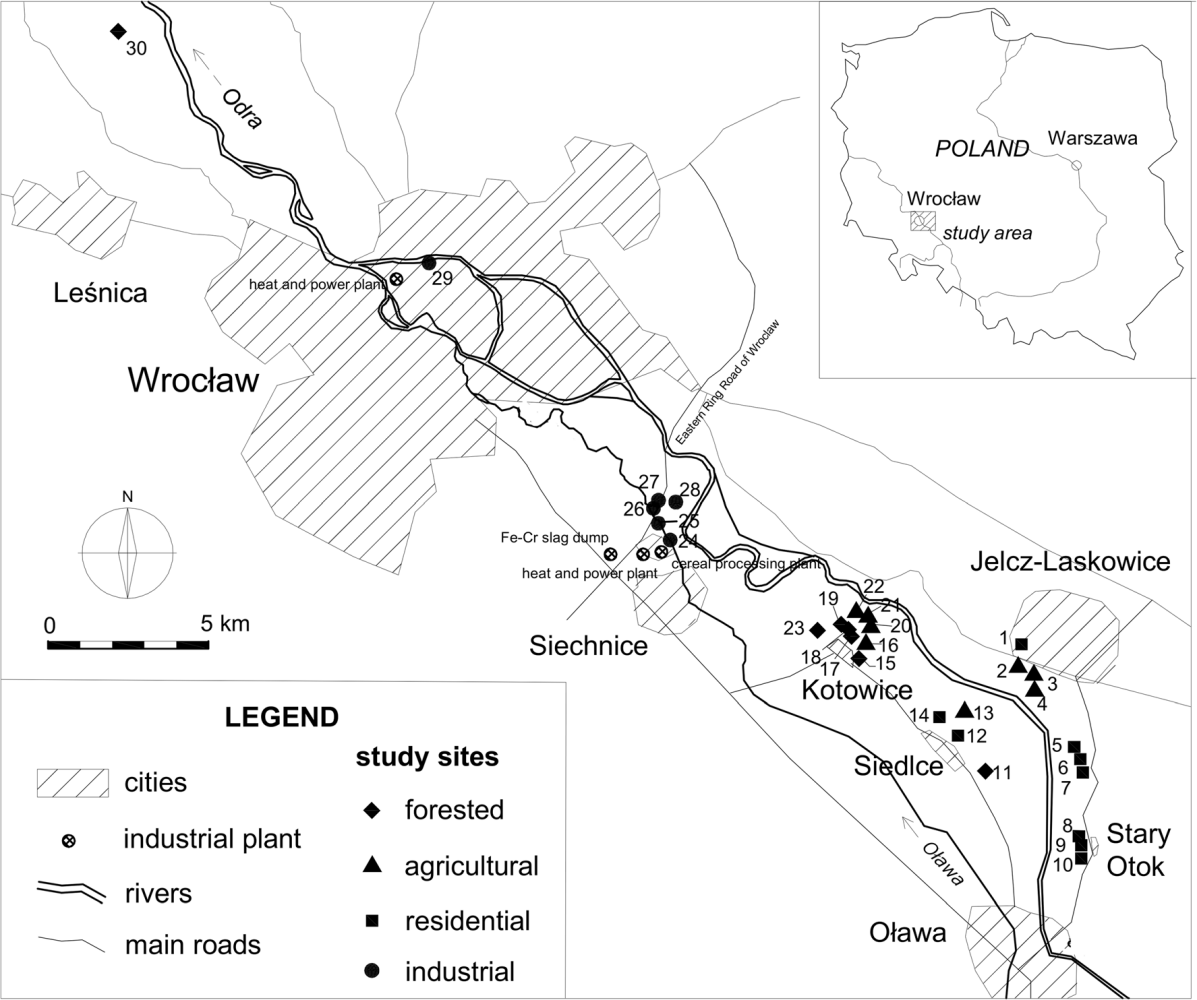
Location of the study sites (•)

The area under study was located in Lower Silesia (SW Poland). Thirty study sites were designated in oxbow lakes, rivers and ponds (Fig. 1). The sampling sites were assigned to four groups according to the dominating type of land use:

1. Forested (numbers 11, 15, 17–19, 23, 30)

Study site number 11 was located near Siedlce, while 15, 17–19 and 23 near Kotowice, villages situated between Siechnice and Oława. The study sites were small water ponds and oxbow lakes located in the inter-embankment of the Odra River and surrounded by forests (riverside ash-elm forest *Ficario-Ulmetum* Knapp) (Fabiszewski 2005). Study site 30 was an old oxbow lake of the Oder, located north-west of Wrocław in a forested area.

2. Agricultural (numbers 2–4, 13, 16, 20–22)

Study sites 2–4 were located in the Matunin oxbow lake between Jelcz-Laskowice and the Odra, while study sites 13, 16 and 20–22 in reservoirs near Siedlce and Kotowice. The villages are located at a distance from the main road (about 3.3–3.8 km). Their surroundings are dominated by wastelands, fields, pastures and scrubs. Some of the reservoirs are also used for angling.

3. Residential (numbers 1, 5–10, 12, 14)

Study site 1 was located in a fish-breeding pond on the edge of Jelcz-Laskowice, a small city. Study sites 5–10 were located along the main road that connects Jelcz-Laskowice and Oława. Study sites number 12 and 14 were located adjacent to buildings in Siedlce. All of these study sites are used by local residents (e.g. for recreation and angling), and additionally may be exposed to runoff from agricultural fields.

4. Industrial (numbers 24–29)

Study sites numbers 24–28 were located in backwaters of the Oława River near Siechnice and study site 29 in the Odra, in an industrial district of Wrocław, near a heat and power plant. Siechnice is a small industrial city, where a ferrochrome steel plant operated until 1995. Nowadays, the main pollution sources are the Czechnica Heat and Power Plant, a slag heap and a few small manufacturing plants, e.g. cereals processing plant, metallurgical plants, concrete plant and logistics centres (Fabiszewski 2005; Meinhardt et al. 2015). Therefore, the region is considered polluted with trace metals and organic compounds (Biłyk and Kowal 1993; Polechońska and Samecka-Cymerman 2016).

### Sample collection

*S. natans* and water samples were collected in corresponding locations in September 2017. In each study site, about 50 g of fresh plants was collected from randomly chosen mono-specific populations with coverage of nearly 100% (Steffenhagen et al. 2012). Subsequently, the plants were washed in reservoir water to remove any sediments and/or invertebrates attached to the surface, and to avoid the introduction of external contamination, the samples were kept in polyethylene bags. Water samples were collected at a depth of 10 cm below the water surface to acid-cleaned polyethylene bottles and stored in a refrigerator. Water pH was measured immediately after collection using a portable pH/conductivity meter (CPC-105, Elmetron).

For phytomass measurements, all plants growing on 1 m^2^ were collected, washed thoroughly in distilled water and oven-dried at 60 °C until constant mass, and then weighed (Vymazal 2016).

### Laboratory analysis

Water samples were filtered through a Whatman glass microfiber filter (GF/C) and acidified to pH ≤ 2 with spectrally pure HNO_3_ (65%, for analysis, Merck KGaA) (Herlich 1990). Fresh plants were rinsed with deionised water to remove external metal ions (Fuentes et al. 2014; Vymazal 2016), dried in a laboratory drier at 60 °C and milled in a POLYMIX PX-MFC 90 D laboratory mill (Kinematica AG, Switzerland). Subsequently, the samples (0.5 g) were digested in an open system in HNO_3_ (5 mL, 65%, for analysis, Merck KGaA) with addition of H_2_O_2_ (1 mL, for analysis, Chempur), whereas temperature was raised to 125 °C until the evolution of nitrous oxide gas stopped and the digest became clear. After cooling to room temperature, the digests were diluted to 50 mL with deionised water and filtered (Piper 1966; Benton Jones Jr. 2001). Trace element (As, Cd, Co, Cr, Cu, Fe, Mn, Ni, Pb, Zn) contents were determined using an atomic absorption spectrophotometer (AAS) (AVANTA PM from GBC Scientific Equipment, Australia) (Karla 1998). Concentrations of As, Cd, Co, Cr and Pb in water were below the detection limits of 0.2 μg L^−1^, 0.0065 μg L^−1^, 0.085 μg L^−1^, 0.01 μg L^−1^ and 0.15 μg L^−1^, respectively.

For quality control, two separate weighed portions (subsamples) of each sample were analysed. Blanks were analysed along with the samples using the same procedures and reagents. All elements were assayed against Sigma Chemical Co. standards. Results were calculated on a dry weight basis. For the evaluation of measurement precision and accuracy, certified reference material WEPAL-IPE-176 (Reed/*Phragmites communis*, WEPAL, Wageningen, The Netherlands) was used (Karla 1998). Recoveries between found and certified values were in the range of 95–104%.

### Data analysis

The normality of data was checked by means of Shapiro-Wilk’s *W*-test, and the homogeneity of variances by means of Levene’s test. To obtain normal distribution of features, Box-Cox transformation was used. Differences between four groups of sampling sites in terms of the concentration of elements in water and plants as well as BF values were evaluated by ANOVA (Zar 1999). The least significant difference (LSD test) was calculated to compare the concentrations of elements (Sokal and Rohlf 2003). A value of *p* < 0.05 was considered statistically significant.

The bioaccumulation factor (BF) was used to evaluate the potential ability of the plant to accumulate elements from water. The BF was computed as follows:

$$ \mathrm{BF}=\frac{C\mathrm{p}}{C\mathrm{w}} $$

*C*p represents element content in the plant (on a dry weight basis) and *C*w is element concentration in water (Ahmad et al. 2016). BF was calculated only for metals detected both in plant tissues and water in all study sites.

The element stock in plants (g m^−2^) was calculated by multiplying the dry biomass yield and element content in the plant (Brezinová and Vymazal 2015; Vymazal 2016).

The calculations were done with CSS Statistica 13.0 software (StatSoft, Inc., STATISTICA data analysis software system).

### Neural network

Neural networks provide a computational method which can be used for visualisation, generation of feature maps, pattern recognition and classification. In the present study, the Kohonen artificial neural network (self-organising feature map, SOFM) (Kohonen 1995) was used to classify water and the plant in terms of trace metal contents to identify clusters of study sites and to identify relationships between similar classes. This form of neural network in which there are no known dependent variables is used in unsupervised clustering (Nisbet et al. 2009). The network transforms high-dimensional data (input layer) into a one- or two-dimensional discretized representation (output layer) simultaneously grouping together map data similarities (Kohonen 1995). This is a “winner takes all” network. During competitive training, the cluster centres are assessed to a radial layer by iteratively submitting training patterns to the SOFM and adjusting the winning unit and its neighbours to the training design. If two inputs are similar, the most active processing elements responding to inputs are located near each other in the map and the weight vectors of the processing elements are arranged in ascending or descending order (Nisbet et al. 2009; Torma 1994). The SOFM overcomes the difficulty of human visualisation of high-dimensional data and accomplishes the reduction of dimensions while at the same time displaying similarities (Kosior et al. 2015).

The structure of the SOFM consisted of two layers of neurons connected by weights (i.e. connection intensities). The input layer consisted of 5 (trace metals that were detectable both in water and plant samples: Cu, Fe, Mn, Ni and Zn) neurons connected to 30 study sites. Kohonen’s network was created in the form of a two-dimensional map. The output layer consisted of 16 neurons (visualised by hexagonal cells) organised in an array with four rows and four columns. Unsupervised training was conducted according to the classical Kohonen algorithm. The net was initiated by the Random-Gauss method. The training consisted of the ordination phase (100 steps/echoes) and the tuning phase (1000 echoes). The winning neuron (i.e. the one nearest to the input case) was selected (Kohonen 1995).

## Results and discussion

Water pH was close to neutral (ranged between 6.87 and 8.93) and in ranges typical for surface waters (Dojlido 1995). The concentrations of elements in water from four groups of study sites are summarised in Table 1. The mean concentrations of all elements except Cu did not exceed typical values noted in clean Polish freshwaters (Dojlido 1995; Kabata-Pendias and Pendias 1999). Only Fe and Mn concentrations in water differed between areas of different land use types (ANOVA, *p* < 0.05) and were significantly higher in industrial and residential areas than the other ones (LSD test, *p* < 0.05).

**Table 1:** Mean, minimum, maximum and standard deviation (SD) of concentration of elements in water in groups of study sites and results of ANOVA with LSD test

	Forested areas (*n* = 7)				Residential areas (*n* = 9)				Agricultural areas (*n* = 8)				Industrial areas (*n* = 6)				GB
	Mean	Min	Max	SD	Mean	Min	Max	SD	Mean	Min	Max	SD	Mean	Min	Max	SD	
Cu (μg L^−1^)	2.41 ^a^	1.02	3.71	1.02	3.36 ^a^	0.15	11.9	3.87	2.71^a^	0.83	6.18	2.22	4.56^a^	1.53	8.48	2.37	2.00*
Fe (μg L^−1^)	35.7^a^	10.6	101	30.7	143^b^	43.8	384	118	41.4^a^	11.5	92.6	27.5	146^b^	27.1	432	145	10–1400**
Mn (μg L^−1^)	128^ac^	16.0	459	161	291^b^	34.0	571	169	55.0^c^	9.00	227	73	176^ab^	43	445	167	1000*
Ni (μg L^−1^)	2.83 ^a^	1.23	4.44	1.03	2.19 ^a^	1.09	3.46	0.80	2.28^a^	1.29	6.21	1.64	2.60^a^	1.32	4.57	1.41	3.00*
Pb (μg L^−1^)		BDL	1.86	0.68		BDL	1.55	0.51		BDL	1.17	0.49	2.39^a^	0.47	4.36	1.48	3.00*
Zn (μg L^−1^)	5.66 ^a^	1.83	12.2	4.24	2.79 ^a^	0.99	7.39	1.92	3.75^a^	0.28	15.04	4.67	4.82^a^	2.43	7.19	1.65	10.0**

GB—geochemical background in Poland: *Dojlido (1995); **Kabata-Pendias and Pendias (1999)

Mean values with the same letter in a row are not significantly different (ANOVA and LSD test, *p* < 0.05)

*BDL* below detection limit (for Pb, 0.15 μg L^−1^)

**Fig. 2:**
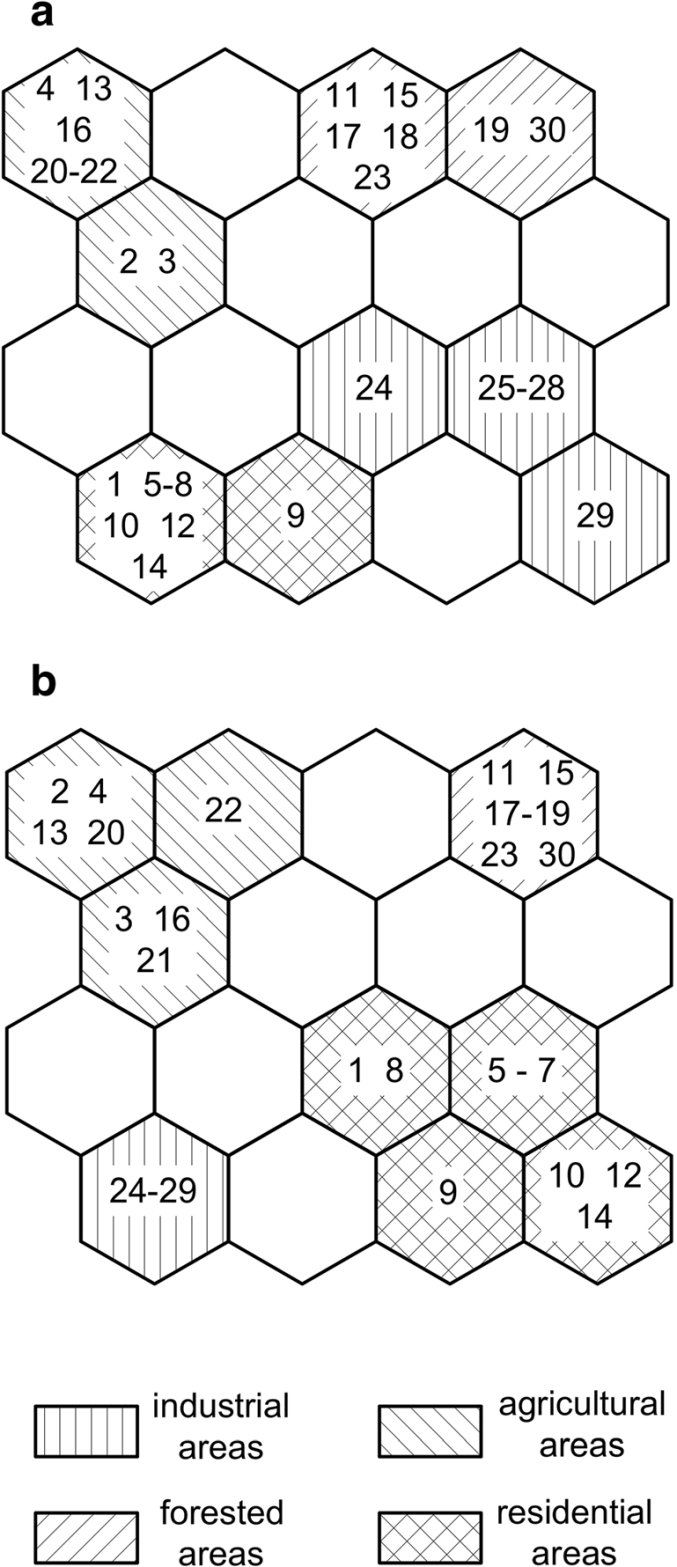
SOFM of trace metal (Cu, Fe, Mn, Ni and Zn) concentrations in water and *Salvinia natans* based on a 4 × 4 Kohonen layer (a: water, b: plants)

In the present investigation, SOFM was used to identify groups of similar study sites in terms of trace metal contents in water and *S. natans* tissues. In the resulting self-organising maps (Fig. 2), each cell represents a neuron. Sampling sites in one cell (neuron) have the most similar features, and the dissimilarity level between the sampling sites grows with the increasing distance between the cells. Both self-organising maps prepared for water and plants clearly showed that cells occupied by samples collected in sites of the same type of land use formed groups. In particular, samples from agricultural areas grouped together and were separated from the groups of samples collected in other anthropogenic areas (industrial and residential). The samples collected in less polluted sites (in forests) also grouped together and were separated from other groups. The neuron clusters for the plants (Fig. 2b) corresponded with the neuron group of the water (Fig. 2a), although in the map classifying water samples two industrial and residential sites were adjacent to each other indicating some level of similarity in trace metal contents. This similarity may be related to similar trace metal sources in industrial and residential areas (e.g. traffic, use of fossil fuels, abrasion of roads, vehicles and buildings). The slight differences between SOFMs for water and plants (Fig. 2 a and b, respectively) should be attributed to fluctuations of contamination, as in waters metals are generally quickly diluted, while plants are indicators of long-term accumulation (Baldantoni et al. 2005). Nevertheless, both maps show the same general trend as well as corresponding groups indicating the relationships between element contents in the plants and their environment. These results confirm that SOFMs are useful in the bioindication of trace element pollution based on the chemical composition of free-flowing plants. SOFMs have been already used in bioindication studies as tools in the classification of the relations between the chemical composition of emergent aquatic macrophytes (Klink et al. 2016; Samecka-Cymerman et al. 2007).

**Fig. 3:**
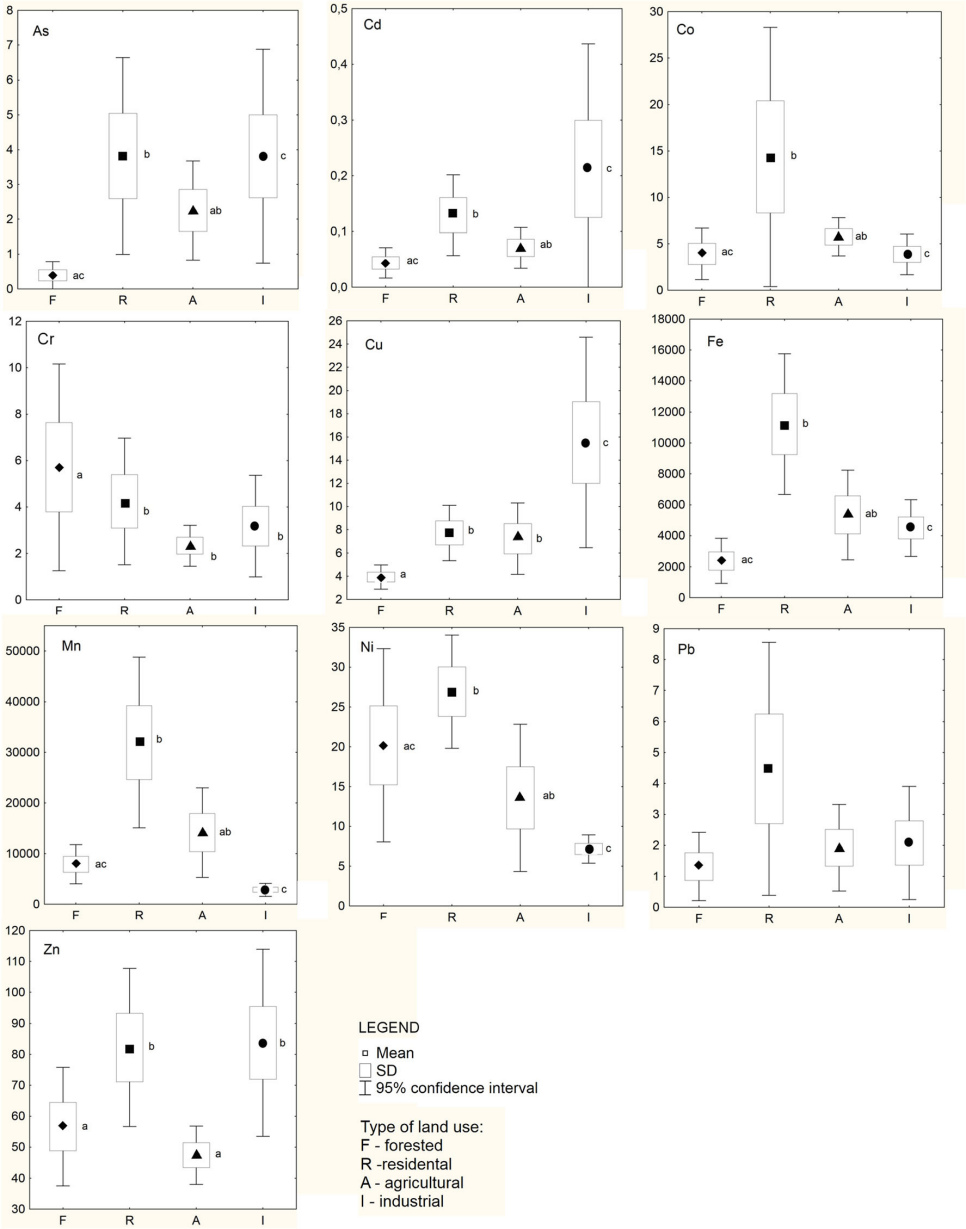
Trace element contents (mg kg^−1^) in *Salvinia natans* from four groups of study sites. The symbols represent mean values; boxes are standard deviation (SD); whiskers are 95% confidence interval. Different letters indicate statistically significant differences between groups (post hoc LSD test, *p* < 0.05)

The results showed by SOFMs were confirmed by statistical analysis. The mean content of all trace elements except Pb differed significantly between plants from groups of study sites characterised by different land use (Fig. 3) (ANOVA, *p* < 0.05). Plants from residential areas had the highest contents of most trace elements (As, Co, Cr, Fe, Mn, Ni, Pb), while plants from industrial areas contained the highest concentrations of Cd, Cu and Zn. Contamination with the trace elements may indicate a wide range of human activities, but the most probable sources in the areas under study are as follows: intensive coal utilisation (e.g. in thermal power plants and as fuel in house heating), traffic as well as abrasion of roads and vehicles (Ali et al., 2013; Kabata-Pendias 2010; Reimann and de Caritat 1998). Moreover, in the industrial areas under study, elevated contents of Cd, Cr, Cu and Zn could have come from spreading waste dust from the ash field and the heap of the former Fe-Cr smelter as well as emissions from the Czechnica Heat and Power Plant (Biłyk and Kowal 1993; Meinhardt et al., 2015). Slightly elevated contents of As, Co, Cr, Cu, Fe, Ni and Mn were also noted in agricultural areas, which could result from use of fertilisers, plant protection products, animal fodder and waste disposal (Ali et al. 2013; Kabata-Pendias 2010; Reimann and de Caritat 1998). The contents of As, Cd, Co, Cr, Cu, Fe, Mn and Pb were the lowest in forested sites (LSD test, *p* < 0.05).

Cobalt, Cu, Mn and Ni contents in *S. natans* were relatively high in all studied locations and exceeded natural contents in freshwater vascular plants (0.32, 7.9, 370, 4.2 mg kg^−1^, respectively) pointing to high accumulation abilities of the species. The Cr content was elevated in agricultural, industrial and residential areas only (Brooks and Robinson 1998). Compared with other aquatic ferns, *S. natans* contained more Fe and Mn than *Azolla japonica* Fr. et Sav. from a landfill area (864 and up to 1470 mg kg^−1^, respectively) (Song et al. 2016), as well as more Cr, Cu, Ni and Zn than *Azolla pinata*R. Br. growing in polluted waters (0.30, 0.9, 0.35, 2.10 mg kg^−1^, respectively) (Bharti and Banerjee 2012; Shafi et al. 2015). What’s more, trace metal contents in *S. natans* were higher than in some free-floating aquatic vascular plants considered hyperaccumulators and used in phytoremediation. In industrial and residential areas, the species contained more Cd and Cu than *Eichhornia crassipes* (Mart.) Solms growing in contaminated waters (0.037–0.13 and 13.5 mg kg^−1^, respectively) (Prasad and Maiti 2016). Mn and Ni contents in *S. natans* from all locations and Cr from all except for forested areas were higher than in *Pistia stratiotes* L. cultivated in stormwater detention ponds in India (760, 7.97 and 3.12 mg kg^−1^, respectively) (Lu et al. 2011). Moreover, the Mn content recorded in *S. natans* was higher than the threshold required for hyperaccumulation, i.e. 1% dry weight; hence, the species can safely be considered a hyperaccumulator for this metal (Dhir et al. 2011).

The bioaccumulation performance of the species (Table 2) was very high and, in case of Cu, Fe, Ni, Mn and Zn, exceeded the criterion established for hyperaccumulators (BF > 10^3^) (Ahmad et al. 2016). The BFs for Cu, Mn and Zn were higher than the average values for freshwater aquatic macrophytes (1128, 52857 and 2600, respectively) (Brooks and Robinson 1998). These results were consistent with laboratory tests that showed a high capacity of *S. natans* for Cu accumulation (Hołtra and Zamorska-Wojdyła 2014). The species was more effective in bioaccumulation compared with other aquatic plants. *S. natans* showed more intensive accumulation of Cu, Fe and Zn (BF = 5000, 100,000 and 10,000, respectively) than *P. stratiotes* (Lu et al. 2011) and, in the case of Mn, than *E. crasspies* and *Pistia* species that are considered effective phytoremediators (Lu et al. 2011; Prasad and Maiti 2016). The Ni BF values in most study sites were higher than for another aquatic fern *Salvinia minima* Baker, which is a hyperaccumulator for Ni (BF 1713-7429) (Fuentes et al. 2014). Although significant differences between areas occurred in case of Fe, Mn and Ni only (ANOVA, *p* < 0.05), the BF values were generally the highest in residential areas confirming intensive bioaccumulation of metals in polluted sites.

**Table 2:** Mean bioaccumulation factor (BF) for elements in *S. natans* in groups of study sites and results of ANOVA with LSD test

	Forested areas	Residential areas	Agricultural areas	Industrial areas
Cu	2081^a^	9526^a^	4744^a^	3884^a^
Fe	56,292^a^	312,224^b^	149,025^c^	128,998^c^
Mn	1,796,567^a^	4,089,961^b^	2,391,957^ab^	100,572^c^
Ni	8790^ab^	13,810^b^	7303^a^	3,289^a^
Zn	16,703^a^	42,438^a^	39,150^a^	21,640^a^

Mean values with the same letter in a row are not significantly different (ANOVA and LSD test, *p* < 0.05)

Element offtake (the quantity of the element removed with harvest), which is an important feature in assessing phytoremediation suitability, depends on element contents and amount of biomass, and element contents in plants only do not provide a full insight into the accumulated amount of elements (Brezinová and Vymazal 2015; Vymazal 2016). The mean biomass of *S. natans* (63.5 g m^−2^ d.w.) was in the range typical for small free-floating species (below 500 g m^−2^ d.w.) (Boyd 1971). The reported value was higher than the biomass of *Lemna minor* L. but lower than that for *Lemna gibba* L. from wetlands of the Tingitan Peninsula (Northwest Africa) (44.5 and 161 g m^−2^, respectively) (Ennabili et al. 1998), and similar to *L. minor* and *Spirodela polyrhiza* (L.) Schleid. from Northeastern Germany (64–69 and 71 g m^−2^ d.w., respectively) (Steffenhagen et al. 2012) as well as *Hydrocharis morsus-ranae* L. in Lower Silesia (60.1 g m^−2^ d.w.) (Polechońska and Samecka-Cymerman 2016). However, the collected biomass was much lower than the average phytomass of *E. crassipes*, a species that is often used in phytoremediation (1275 g m^−2^ d.w.) (Rezania et al. 2016; Wetzel 2011). On the other hand, the species showed Cd, Co, Cr, Cu, Mn, Ni and As accumulation rates (Table 3) significantly higher than those reported for *Phragmites australis* (Cav.) Trin. ex Steud. and *Phalaris arundinacea* L. commonly used in constructed wetlands (Bernard and Lauve 1995; Brezinová and Vymazal 2015; Caçador et al. 2009; Kumwimba et al. 2017; Maddison et al. 2009). Thus, despite the relatively small biomass, harvesting *S. natans* may have an influence on the removal of trace elements from aquatic ecosystems.

**Table 3:** Average element stock (mg m^−*2*^) for elements in *S. natans*

Element	As	Cd	Co	Cr	Cu	Fe	Mn	Ni	Pb	Zn
Element stock	0.16	7.24 × 10^−3^	0.44	0.26	0.55	372	902	1.08	0.16	4.29

## Conclusions

Differences of trace element contents in *S. natans* between areas with different land use point to the possibility of using the plant in bioindication. Industrial areas had the highest content of Cd, Cu and Zn in plants while residential areas had the highest content of As, Co, Cr, Fe, Mn, Ni and Pb. Trace element contents in plants in forested areas were generally the lowest. Application of SOFMs confirmed that trace element accumulation in plants corresponds to the concentrations of elements in water and type of land use.

The plant may be considered accumulator for Cu, Fe, Ni and Zn and hyperaccumulator for Mn. In spite of a relatively low biomass, *S. natans* may have an influence on the removal of trace elements from aquatic ecosystems due to a large element stock, especially of Cd, Mn and Ni. In connection with the high growth rate and easy harvesting of the species, these features make *S. natans* a potential candidate for phytoremediation. However, the use and manipulation of the species would require special care, as it has some expansive characteristics and its control may be problematic in natural reservoirs.
